# Relation between urinary hydration biomarkers and total fluid intake in healthy adults

**DOI:** 10.1038/ejcn.2013.93

**Published:** 2013-05-22

**Authors:** E Perrier, P Rondeau, M Poupin, L Le Bellego, L E Armstrong, F Lang, J Stookey, I Tack, S Vergne, A Klein

**Affiliations:** 1 Danone Research, Palaiseau, France; 2 Human Performance Laboratory, University of Connecticut, Storrs, CT, USA; 3 Institute of Physiology, University of Tübingen, Tübingen, Germany; 4 Children's Hospital Oakland Research Institute, Oakland, CA, USA; 5 Department of Clinical Physiology, Toulouse Hospital and University, Toulouse, France

**Keywords:** osmolality, urination, hydration, beverages, water, intake

## Abstract

**Background/objectives:**

In sedentary adults, hydration is mostly influenced by total fluid intake and not by sweat losses; moreover, low daily fluid intake is associated with adverse health outcomes. This study aimed to model the relation between total fluid intake and urinary hydration biomarkers.

**Subjects/methods:**

During 4 consecutive weekdays, 82 adults (age, 31.6±4.3 years; body mass index, 23.2±2.7 kg/m^2^; 52% female) recorded food and fluid consumed, collected one first morning urine (FMU) void and three 24-h (24hU) samples. The strength of linear association between urinary hydration biomarkers and fluid intake volume was evaluated using simple linear regression and Pearson's correlation. Multivariate partial least squares (PLS) modeled the association between fluid intake and 24hU hydration biomarkers.

**Results:**

Strong associations (|*r*|⩾0.6; *P*<0.001) were found between total fluid intake volume and 24hU osmolality, color, specific gravity (USG), volume and solute concentrations. Many 24hU biomarkers were collinear (osmolality versus color: *r*=0.49–0.76; USG versus color: *r*=0.46–0.78; osmolality versus USG: 0.86–0.97; *P*<0.001). Measures in FMU were not strongly correlated to intake. Multivariate PLS and simple linear regression using urine volume explained >50% of the variance in fluid intake volume (*r*^2^=0.59 and 0.52, respectively); however the error in both models was high and the limits of agreement very large.

**Conclusions:**

Hydration biomarkers in 24hU are strongly correlated with daily total fluid intake volume in sedentary adults in free-living conditions; however, the margin of error in the present models limits the applicability of estimating fluid intake from urinary biomarkers.

## Introduction

Water is essential for a wide variety of physiological functions, and water intake must be sufficient to compensate for daily losses. Low daily water intake and low urine output increase long-term risk of kidney dysfunction,^1, 2^ and insufficient intake may also have a role in the development of hyperglycemia.^3^ Guidelines for adequate total water intake for the general adult population have been proposed by several international governing bodies.^4, 5^ Yet, these recommendations are based on population median water intakes, with limited consideration of links between water intake and hydration status and without links between water intake and health. Moreover, the recommendations do not provide a method for individuals to ensure they are consuming enough water to meet their specific hydration needs.

Establishing the adequacy of fluid intake based on physiological indicators of hydration is challenging, because there are multiple biological indicators of hydration in average adults in free-living conditions, each sensitive to a different aspect of hydration. The accurate measurement of hydration status is complicated because body water turnover occurs constantly and water moves between intracellular and extracellular compartments. Proposed biomarkers of hydration include the direct measurement of blood, urine, saliva and tears^6, 7, 8, 9, 10^ as well as estimates of body water via bioelectrical impedance or spectroscopy. No single method appears to be ideal for all situations.^11, 12, 13^ Serum osmolality is considered a good marker for acute or critical dehydration situations,^6, 7^ but is tightly regulated and insensitive to mild hydration deficits in healthy sedentary individuals with ad libitum access to fluids.^5, 14^ In contrast, urinary hydration biomarkers do vary according to fluid intake,^14, 15, 16^ and are biologically significant in terms of predicting health outcomes such as chronic kidney disease.^1^

Recent work by Armstrong *et al.*^15, 16^ established reference ranges for various urinary and plasma hydration biomarkers relative to deciles of total water intake volume; this suggests the possibility of a linear association between intake and hydration biomarkers. If strong linear relations exist between urinary hydration biomarkers and fluid intake, it may be possible to link daily fluid intake volume to biomarkers that are easily measured. This would represent an important advancement in the ability to monitor daily fluid intake, hydration and disease risk, and may help individuals determine a daily fluid intake volume appropriate for their individual hydration requirements.

Our research group recently published an assessment of the impact of habitually different total fluid intake behaviors (low consumption: <1.2 l per day versus high consumption: >2.0 l per day) on multiple biomarkers of hydration status in free-living conditions.^14^ Hydration biomarkers in first-morning and 24-h urine (24hU) collections, but not plasma osmolality, were significantly different between low and high volume drinkers, and urine osmolality, volume and specific gravity (USG) were distributed over a broad range of values. The study database also included individuals whose fluid consumption fell between the established low and high drinker thresholds, and who were therefore excluded from the previous low/high analysis. This presented us with an opportunity to further explore the associations between hydration biomarkers and total fluid intake over a wide range of fluid intake behaviors. The purpose of this analysis was to examine the strength of correlations between total daily fluid intake and urinary hydration biomarkers in sedentary adults in free-living conditions, and to model fluid intake relative to urinary hydration biomarkers using simple and multivariate regression methods.

## Materials and methods

### Subjects

The participant recruitment and study design have previously been described.^14^ A total of 82 healthy adults (age: 31.6±4.3 years; body mass index, 23.2±2.7 kg/m^2^; 52% female) were included in this analysis. Inclusion criteria included the ability to stay at home and abstain from strenuous physical activity, access the internet and live within 30 min of the investigating center. Exclusion criteria included use of medication likely to interfere with water balance, such as hypotensive or diuretic treatment; history of metabolic or gastrointestinal disease; renal, hepatic or cardiac failure, smoking more than 15 cigarettes daily; or high daily consumption of alcohol (more than 2 units or 3 units per day for women and men, respectively). The study was conducted according to the guidelines set forth in the Declaration of Helsinki, and all procedures involving human subjects were approved by the Comité de Protection des Personnes of Ile de France XI. Written informed consent was obtained from all subjects.

### Experimental design

This observational study evaluating fluid intake and urinary hydration biomarkers was carried out over the course of four consecutive evaluation visits scheduled Tuesday through Friday. Weekends were excluded to limit within-person day-to-day variation. Enrolled subjects were asked to eat and drink as usual, and record all food and fluid consumption using an online diary (e-diary; MXS Epidémio, France). Food and fluid item selections were made from a reference databank listing the nutritional composition of more than 1300 generic foods.^17^ Beginning on Monday morning, subjects used the e-diary to record all food and fluid intake through Thursday night. Tuesday morning, subjects collected their first morning urine (FMU) at home and delivered it to the clinic. Study participants returned to the clinic over the next three consecutive mornings to deliver 24hU collections (24hU-1, 24hU-2, 24hU-3), which were checked for completeness.^14^

Daily total fluid intake was estimated from the e-diary. Urine osmolality was determined via freezing point depression osmometry (Messtechnik; Giessen, Germany), USG via a digital refractometer (ATAGO Co.; Tokyo, Japan) and volume was calculated from urine mass and USG. Urine color was determined using the 8-point scale developed by Armstrong *et al.*^18, 19^ All urine was stored at +4 °C and the central laboratory remained blinded to the fluid intake of the participants.

### Statistical analysis

Pearson's correlation coefficients were calculated to determine the strength of the relationship (1) between each recorded 24 h fluid intake volume and associated 24hU hydration biomarkers, and (2) between 24hU hydration biomarkers. The decision was made *a priori* to identify only strong relationships (that is, |*r*|⩾0.6) between urinary hydration biomarkers and total fluid intake.

Next, a multivariable partial least squares (PLS) model of fluid intake as a function of 24hU biomarkers was developed to identify key predictors in modeling total fluid intake. The PLS model took into account 17 urinary biomarkers (urine volume, osmolality, pH, color and USG; concentrations of sodium, calcium, uric acid, cortisol, citrate, potassium, phosphate, urea, aldosterone, magnesium, creatinine and oxalate) as predictors of total fluid intake. The relative contribution of each predictor in fitting the model (variable importance for projection) was calculated for each (Table 1), using a cutoff of variable importance for projection>0.8 (SAS Institute Inc.; SAS version 9.2, Cary, NC, USA).

Given that a model requiring a single measure would be most practical for widespread use, urinary hydration biomarkers with a variable importance for projection>1.20 were identified. These urinary biomarkers were used to generate simple linear regression models of fluid intake for comparison with the full PLS model. Using fluid intake and urinary data from a single collection day, the predicted versus observed values for total fluid intake generated from the simple linear and PLS models were assessed. Limits of agreement between observed and predicted total fluid intake were calculated using the Altman-Bland procedure.

## Results

### Total fluid intake

Mean total fluid intake was 1604±937 ml per day. Within each subject, the lowest and highest reported daily fluid intakes differed by 224±200 ml per day and 219±179 ml per day, respectively, from the within-subject mean. The largest contributor to total fluid intake was plain water, representing 61±27% of total fluid intake. Other beverage types that accounted for more than 10% of total fluid intake were hot beverages (14±19%) and sweetened beverages (12±19%). Combined, milk, diet beverages, flavored water and alcohol made up the remaining 13% of fluid intake.

### Correlations between total fluid intake and urine biomarkers

Strong relations were apparent between multiple 24hU hydration biomarkers and fluid intake (Table 2). Urine volume increased in proportion to fluid intake volume, whereas osmolality, USG and concentrations of uric acid, urea, creatinine, phosphate, sodium and potassium decreased with higher fluid intake. Urine color also was inversely related to fluid intake. Biomarkers in FMU were not strongly correlated with fluid intake.

### Correlations between urine biomarkers

Strong positive correlations were found between measures of 24hU concentration. Pearson's correlation coefficients between osmolality and USG were strong on all days (*r*=0.86, 0.97 and 0.97, respectively, for 24hU-1, 24hU-2 and 24hU-3). Similar associations were noted between osmolality and color (*r*=0.70, 0.49, 0.76), and USG and color (*r*=0.68, 0.46, 0.78). 24hU volume was inversely related to the measures of concentration (*r*=−0.80 for osmolality, −0.78 for USG and −0.70 for color during 24hU-3).

### PLS and simple linear regression

**Figure 1:**
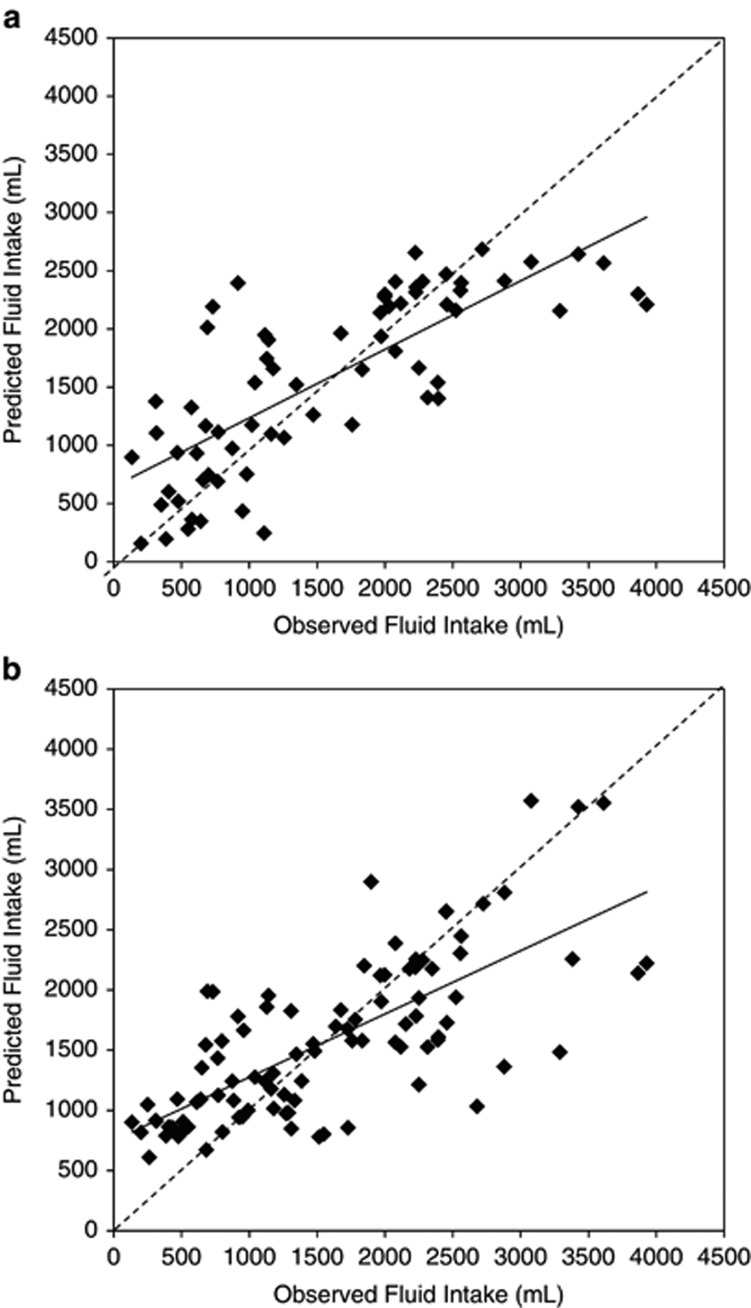
Modeling the relationship between total fluid intake and 24hU hydration biomarkers. Dashed lines represent the line of agreement, while solid lines represent the line of best fit. (**a**) multivariate PLS: *r*^2^=0.59, RMSE=663 ml; (**b**) simple linear regression using urine volume only: *r*^2^=0.52, RMSE=629 ml.

A PLS model of the relationship between total fluid intake and urinary biomarkers was developed using 17 urinary measurements as predictor variables. The percentage of variance in total fluid intake (*R*^2^) explained by the one-factor PLS model was 59% (Figure 1a), with a root mean square error of 663 ml and 95% limits of agreement of (−1231;1231 ml). Sixty-six percent of predicted fluid intake volumes fell within 500 ml of the recorded intake. From the PLS model, two urinary biomarkers were identified as possible key predictors of total fluid intake. 24hU volume and osmolality contributed most heavily to the PLS model (variable importance for projection of 1.28 and 1.22, respectively). Using simple linear regression with 24hU volume as a singular predictor (Figure 1b), the percentage of variance in total fluid intake explained by the model was slightly lower (*R*^2^=52%), but with a comparable root mean square error (629 ml) and 95% limits of agreement (−1238;1238 ml). Similar to the PLS model, 63% of predictions fell within 500 ml of recorded fluid intake. When simple linear regression was repeated using 24hU osmolality as a predictor of total fluid intake, a slightly higher root mean square error (701 ml) and a lower *R*^2^ (41%) resulted.

## Discussion

Recently, our research group published a comparative analysis of urinary and plasma hydration biomarkers in sedentary individuals. We identified significant differences for urinary hydration biomarkers, but no differences in plasma osmolality, between individuals who habitually consume low versus high daily fluid volumes in free-living conditions.^14^ The present investigation explored a second perspective on the relation between 24-h hydration biomarkers and fluid intake by examining the strength of the correlations between urinary biomarkers of hydration and daily fluid intake volume. The results of this analysis suggest that: (1) hydration biomarkers in 24 h, but not FMU collections are strongly correlated with concurrent fluid intake volume; (2) several 24hU biomarkers, notably osmolality, USG, color and volume, demonstrate a high degree of collinearity across a broad range of values; (3) urine volume alone provides information about total water intake with accuracy comparable to multiple variables combined; and (4) both the simple and complex models of total fluid intake in the current study have a large margin of error that limits the ability to accurately estimate total fluid intake volume.

Previous studies of urinary hydration biomarkers have shown that during acute progressive dehydration, USG, urine osmolality and urine color may be used interchangeably to track hydration status.^18, 19^ In sedentary to moderately active adults, where sweat losses are not as pronounced, water balance is largely determined by the adequacy of fluid intake. Our results extend the utility of urinary hydration biomarkers beyond acute water loss and progressive dehydration into the context of drinking behaviors seen in real-life conditions. Within the context of free-living French adults, 24-h USG, osmolality, volume and color were all strongly related. This finding is not surprising, given that urine concentration and volume are inversely related, and that USG, osmolality and color all represent urine concentration. Perhaps more important, the values for these biomarkers span a sufficiently broad range to show linear relations to fluid intake. This suggests that urinary biomarkers of hydration may have the potential to be used to approximate fluid intake volume in situations where collecting fluid intake data is difficult. Moreover, fluid intake records alone account for only one side of the water balance equation, and cannot account for daily water loss. Urinary biomarkers of hydration status, in contrast, provide information about the adequacy of fluid intake relative to body water losses, and are thus more indicative of adequate hydration status than relying on fluid intake alone. In the current study, data was collected during temperate environmental conditions, and the physical activity of participants was restricted to sedentary activities, thus limiting water losses. Thus, we anticipated that urine biomarkers would closely reflect fluid intake volume to the extent possible in a study conducted in free-living conditions.

Currently, adequate intake guidelines for water are based largely on median intake reported in large population surveys. Although collection methods vary, intake studies commonly employ recall-based methods such as 24-h diet recall and food frequency questionnaires that have a tendency to underestimate intake.^20^ Moreover, these instruments were developed mainly to examine nutrient intakes from food; little is known about their validity to accurately assess fluid intake. Despite the shortcomings of dietary intake surveys, water intake guidelines rely heavily on these reported intake values, and rarely take into account the impact of a given level of fluid intake on hydration physiology or health. The ability to model the relationship between fluid intake and urinary hydration biomarkers would allow for the establishment of adequate intake recommendations based on physiological indicators of hydration; moreover, it would facilitate studies evaluating the link between adequate fluid intake and health.

To construct a preliminary model of fluid intake relative to urinary hydration biomarkers, two types of regression analyses were performed. The first, a PLS model that incorporated 17 different urine biomarkers determined which urine biomarkers were relatively important in modeling the relation between urine characteristics and fluid intake. The PLS model also was selected because of its ability to recognize and interpret the high degree of collinearity between biomarkers of urine concentration. The PLS model was able to account for nearly 60% of the variance in fluid intake, with a RMS error of >600 ml, and revealed that 12 of the 17 24hU biomarkers made relatively important contributions to modeling fluid intake. With the mean daily fluid intake in our study of ∼1700 ml, a mean error of >600 ml represents quite a large degree of inaccuracy. Given the time, effort and expertise required to assess many urine parameters, including concentrations of individual solute components, the inaccuracy of the model cannot justify so many measures, particularly in the context of a large study. Moreover, many of the urine parameters included in the model are highly collinear, suggesting that a greatly reduced model may be feasible as well as clinically favorable. Therefore, we also repeated the analysis using simple linear regression against a single urine variable to determine whether the simplest model was comparable in terms of accuracy. Regression of fluid intake against 24hU volume provided essentially the same results as the complex PLS model. The margin of error associated with both the PLS and simple regression models of fluid intake volume remains high. Nonetheless, this model represents a first step toward the possibility of associating intake volume with urinary hydration biomarkers.

Finally, the strong relation between fluid intake and urinary hydration biomarkers, notably 24hU osmolality, provides an opportunity to revisit the opinion of the European Food Safety Authority (EFSA) in establishing its total water intake guidelines for adults (2.5 and 2.0 l per day of total water for men and women, respectively, with ∼80% coming from fluids). EFSA bases its water daily reference values on observed intakes as well as relative to a ‘desirable' urine osmolarity of 500 mOsm/l,^4^ calculated from the renal solute load derived from European dietary surveys. The EFSA recommendations incorporated a calculation of the theoretical amount of water required to excrete solute at a urine osmolarity of <500 mOsm/l. Osmolarity (expressed in mOsm/l) and osmolality (expressed in mOsm/kg) are not synonymous; however, the difference between the two is negligible in solutions of low concentration.^21^ Thus, our study data provided an opportunity to test the cutoff of 500 mOsm/kg relative to daily fluid intake. Overall, the calculations and recommendations made by EFSA were well adapted to the majority of adults in our sample. On days when subjects met or exceeded EFSA reference value for water from fluid, urine osmolality was <500 mOsm/kg 80% of the time. Similarly, urine osmolality exceeded 500 mOsm/kg in 82% of the cases where fluid intake was inferior to daily recommendations. It is worth noting, however, that the reference values for daily water intake may not be sufficient to ensure a low urine osmolality in ∼20% of the population, which is nonetheless substantial. This underscores the large interindividual variability in daily water needs, as well as the necessity of developing biomarkers for adequate fluid intake that are tailored to physiological indicators.

In conclusion, hydration biomarkers strongly related to fluid intake may have utility for epidemiological studies linking adequate fluid intake to health outcomes. Linear relationships between urinary hydration biomarkers and acute, progressive dehydration are well established; our findings extend the validity of urinary hydration biomarkers to average adults with known habitual fluid intake. 24hU parameters (osmolality, USG, color and volume) demonstrate strong linear relations to fluid intake, within a broad range of intake volumes that are exhibited by average adults under normal living conditions. Furthermore, urine osmolality, USG and color were strongly related. When ease of measurement is taken into account, USG and urine color appear to be good candidates to track hydration in normal daily living conditions. However, the regression models examined in the current study do not yet provide a sufficient degree of accuracy to approximate intake from hydration markers alone, as the margin of error in both models was large relative to total fluid intake volume. Nonetheless, the strong relation between urinary hydration and fluid intake may provide a physiological basis for individuals and health care providers to assess whether fluid intake is sufficient to meet individual needs.

## Figures and Tables

**Table 1:** VIP coefficients for 24hU hydration biomarkers in the PLS model

*VIP>0.8*		*VIP<0.8*	
Volume	1.28	[Cortisol]	0.79
Osmolality	1.22	[Calcium]	0.78
[Phosphate]	1.19	[Aldosterone]	0.69
[Uric acid]	1.18	[Oxalate]	0.68
[Urea]	1.16	pH	0.06
USG	1.15		
[Creatinine]	1.13		
[Potassium]	1.11		
[Sodium]	1.05		
[Magnesium]	1.04		
Color	0.95		
[Citrate]	0.82		

Abbreviations: 24hU, 24-h urinary; PLS, partial least squares; USG, specific gravity; VIP, variable importance of projection.

**Table 2:** Correlations between 24-h urinary (24hU) hydration biomarkers and total fluid intake

	*Correlation with total fluid intake*			
	*FMU*	*24hU-1*	*24hU-2*	*24hU-3*
Volume	0.30	**0.79**	**0.74**	**0.78**
USG	−0.33	−0.56	**−0.65**	**−0.70**
Osmolality	−0.43	**−0.71**	**−0.66**	**−0.74**
Color		**−0.61**	−0.42	−0.58
[Sodium]	−0.36	−0.59	−0.49	**−0.64**
[Potassium]	−0.22	−0.59	−0.49	**−0.68**
[Phosphate]	−0.38	**−0.67**	**−0.62**	**−0.73**
[Creatinine]	−0.40	**−0.70**	**−0.63**	**−0.69**
[Uric acid]	−0.36	**−0.70**	**−0.66**	**−0.72**
[Urea]	−0.38	**−0.70**	**−0.65**	**−0.71**

Abbreviations: FMU, first morning urine; USG, specific gravity. Bold values indicate *P*<0.001.

## Notes

EP, PR, MP, LLB, SV and AK are employees of Danone Research. LEA, FL, JS and IT are occasional consultants for Danone Research.
